# FusDRM-m5C: a hybrid model for accurate prediction of 5-methylcytosine modification sites based on feature fusion and attention mechanism

**DOI:** 10.3389/fgene.2025.1642286

**Published:** 2025-12-01

**Authors:** Hui Huang, Fenglin Zhou, Jianhua Jia

**Affiliations:** School of Information Engineering, Jingdezhen Ceramic University, Jingdezhen, China

**Keywords:** m5C site identification, multi-feature fusion, deep learning, dilated convolutional neural networks, multi-head self-attention

## Abstract

**Introduction:**

The precise identification of 5-methylcytosine (m5C), an epitranscriptomic modification fundamental to RNA function, is crucial yet proves difficult to achieve experimentally. Consequently, computational prediction offers a promising avenue; however, refining its predictive accuracy and ensuring its robustness remain ongoing objectives. To address these limitations, this study introduces a deep learning framework designed for highly accurate m5C site prediction from RNA sequences.

**Methods:**

We propose FusDRM-m5C, a deep learning framework featuring a multi-branch architecture designed to process three distinct feature types: one-hot vector representation (one-hot), Z-curve-based geometrical features (Z-curve), and local RNA secondary structure (RSS). Each feature type is processed by a separate, parallel branch. Within each branch, a Dilated Convolutional Neural Network (DCNN) captures multi-scale patterns, followed by a Multi-Head Self-Attention (MHSA) mechanism with residual connections to weigh context-dependent information. For feature fusion, the high-level representations from the three branches are then integrated via concatenation. This fused feature vector is subsequently fed into a final fully connected network, which generates the prediction probability for precise m5C site identification.

**Results:**

The performance of FusDRM-m5C was rigorously evaluated using both 5-fold cross-validation (CV) and independent dataset testing. On the 5-fold CV benchmark dataset, the model achieved high predictive accuracy, reflected by a Sensitivity (Sn) reaching 0.995, Specificity (Sp) of 0.971, Accuracy (ACC) at 0.983, Matthews correlation coefficient (MCC) measuring 0.966, and an Area Under the Receiver Operating Characteristic Curve (AUC) of 0.997. Crucially, when assessed on an independent test dataset, the model maintained strong generalization capability, attaining an Sn of 0.900, Sp of 0.965, Acc of 0.933, MCC of 0.867, and an AUC of 0.986. Furthermore, we assessed the cross-species prediction performance of FusDRM-m5C. The results demonstrated that the model consistently maintained high accuracy and robustness across datasets from multiple species, outperforming several existing state-of-the-art methods.

**Discussion:**

The proposed FusDRM-m5C model demonstrates highly accurate and robust prediction of m5C sites, comparing favorably with existing methods. Its architecture effectively integrates diverse biological features through distinct processing pathways fused via attention, offering a powerful tool for m5C identification.

## 1 Introduction

RNA modifications represent a crucial layer of post-transcriptional gene regulation, influencing various aspects of RNA metabolism including stability, splicing, nuclear export, localization, translation efficiency, and interactions with RNA-binding proteins (Roundtree et al., 2017; Zaccara et al., 2023). Among over 170 known types of RNA modifications (Cappannini et al., 2024), 5-methylcytosine (m5C) has gained increasing attention due to its widespread occurrence and functional significance. m5C has been identified in multiple RNA species, including messenger RNA (mRNA), transfer RNA (tRNA), ribosomal RNA (rRNA), and long non-coding RNA (lncRNA) (Squires et al., 2012; Hussain et al., 2013). Its presence has been associated with diverse biological processes, such as stem cell differentiation, cellular stress response, embryonic development, and tumorigenesis (Blanco et al., 2014; Yang et al., 2017). Therefore, accurate and comprehensive identification of m5C sites is crucial for elucidating its biological roles and understanding its involvement in both normal physiology and disease pathogenesis.

To detect m5C modifications at single-nucleotide resolution, several high-throughput experimental techniques have been developed, including bisulfite sequencing (BS-seq) (Frommer et al., 1992), 5-azacytidine–mediated RNA immunoprecipitation (Aza-IP) (Masiello and Biggiogera, 2017), and methylation individual-nucleotide resolution crosslinking and immunoprecipitation (miCLIP) (Edelheit et al., 2013). These methods have greatly advanced our understanding of m5C distribution and function in various transcriptomes. However, they still suffer from several practical limitations. For example, BS-seq is prone to false positives due to incomplete conversion or structural resistance, Aza-IP and miCLIP require specialized reagents and antibodies, and all these methods typically demand large quantities of high-quality RNA input. Furthermore, the dynamic, tissue-specific, and condition-dependent nature of RNA methylation makes it challenging to achieve comprehensive and consistent experimental profiling across diverse biological contexts.

In response to these challenges, computational approaches have emerged as effective and scalable alternatives to assist in m5C site discovery. By exploiting known sequence characteristics, structural signals, and evolutionary conservation, machine learning–based and deep learning–based models can predict putative m5C sites from RNA sequence data. These computational tools represent a significant improvement over labor-intensive wet-lab approaches, primarily by delivering superior speed and cost-effectiveness. Furthermore, they enable the efficient analysis of large-scale transcriptomic datasets, thereby circumventing the need for supplementary experimental procedures. Importantly, computational methods also enable hypothesis generation for functional studies, guide experimental validation, and facilitate the annotation of RNA methylomes in species or cell types where experimental data are scarce.

Following the initial experimental challenges, the development of computational predictors for m5C sites began to gain momentum. Feng et al. pioneered this area by proposing m5C-PseDNC (Feng et al., 2016), a support vector machine (SVM)-based model that utilized pseudo-K-tuple nucleotide composition (PseKNC) to identify m5C sites in *Homo sapiens*. Subsequently, Qiu et al. introduced iRNAm5C-PseDNC (Qiu et al., 2017), which employed random forests (RF) and incorporated physicochemical properties into PseDNC. In 2019, Fang et al. presented RNAm5CPred (Fang et al., 2019), an SVM-based model that compared balanced and imbalanced datasets while utilizing KNF, K-spaced nucleotide pair frequency (KSNPF), and PseDNC. The field continued to diversify in 2020, with Lv et al. developing iRNA-m5C (Lv et al., 2020), an RF-based predictor for four species that combined PseKNC, MNBE, KNFC, and natural vector (NV) features and also offered a web server. In the same year, Chen et al. introduced m5CPred-SVM (Chen et al., 2020), focusing on three species and innovatively incorporating position-specific propensity features (PSNP, PSDP) along with KNF, KSNPF, PseDNC, and chemical property with density (CPD), also making it available via a web server. Dou et al. further contributed to the development of iRNA-m5C_SVM (Dou et al., 2020) for A. thaliana, an SVM-based model that combines position-specific propensity (PSP), k-mer, pseudo electron-ion interaction potentials (PseEIIP), and pseudo dinucleotide composition (PCPseDNC).

The application of more advanced machine learning techniques and deep learning began to emerge prominently thereafter. Chai et al. introduced Staem5 (Chai et al., 2021), employing a stacking ensemble model with various base classifiers for A. thaliana and *M. musculus*. Concurrently, the exploration of deep learning intensified, with Hasan et al. proposing Deepm5C (Hasan et al., 2022), a deep learning-based hybrid framework that utilizes a stacking strategy for human m5C site identification. Liu et al. developed m5Cpred-XS (Liu et al., 2022), leveraging XGBoost and SHAP for feature interpretation and prediction in *H. sapiens*, *M. musculus*, and A. thaliana. Xiao et al. focused on m5C sites in human promoters using m5C-HPromoter (Linghui et al., 2022), an ensemble deep learning approach that employs frequency-based One-Hot encoding. A unique contribution was made by He et al. with m5CRegpred (He et al., 2022), which addressed the substrate specificity of m5C writers and readers.

More recent advancements have continued to push the boundaries of deep learning applications. Aslam et al. proposed a 1D Convolutional Neural Network (CNN) based predictor (Aslam et al., 2023). Malebary et al. further explored deep learning architectures such as GRU, LSTM, and Bi-LSTM in their m5c-iDeep model (Malebary et al., 2024). Kurata et al. developed MLm5C (Kurata et al., 2024), a high-precision predictor that utilizes a stacking ensemble of hybrid machine learning models and evaluates a broad set of 11 distinct features. Transformer-based architectures, known for their success in natural language processing, were introduced to this domain by Fu et al. with Trans-m5C (Fu et al., 2025). Jiang et al. enhanced prediction accuracy in Feadm5C (Jiang et al., 2025) by incorporating physicochemical molecular graph features with BiLSTM. Concurrently, efforts to refine k-mer-based approaches and ensemble strategies continued, as evidenced by m5C-TNKmer (Qazi et al., 2025) and m5c-iEnsem (Bilal et al., 2025), which focused on bagging and boosting techniques. These collective efforts highlight a continuous drive towards more accurate, robust, and interpretable computational models for m5C site prediction across diverse species and biological contexts.

Although these approaches have significantly advanced m5C prediction, several limitations remain. Many models rely heavily on sequence-derived features while underutilizing the RNA structural context. Certain methods are hindered by architectural designs that offer insufficient representational capacity, consequently impeding their ability to capture the long-range dependencies crucial for precise prediction. Moreover, a number of current tools demonstrate suboptimal predictive performance, especially when evaluated on unseen datasets, which clearly indicates difficulties in generalization. Cross-species applicability and model interpretability also remain open issues.

To address the limitations of current m5C site prediction methods, we propose FusDRM-m5C, a hybrid deep learning framework. The main contributions of this study are as follows:

1. FusDRM-m5C adopts a tailored deep learning architecture that integrates multiple neural components to effectively capture distinct biological features. By combining dilated 1D convolutions for modeling long-range dependencies, 2D convolutions for extracting spatial patterns from RNA secondary structure, and multi-head self-attention for enhancing global contextual awareness, the model ensures comprehensive and stable feature extraction. Residual connections further support training convergence and the flow of information.
2. The model’s multi-feature fusion architecture processes sequence (one-hot, Z-curve) and secondary structure inputs through parallel channels. Each channel’s output is first refined by a dedicated multi-head attention mechanism before the representations are concatenated and fed to a fully connected network. This method ensures an effective integration of diverse biological information, leading to enhanced predictive accuracy.
3. FusDRM-m5C achieved competitive results on diverse benchmark datasets. In both five-fold cross-validation and independent testing, the model showed improved performance over several existing approaches in key metrics including AUROC and accuracy, which suggests its potential effectiveness and reliability in m5C site prediction.
4. The proposed model showed promising generalization capabilities in cross-species validation tasks. The results indicated high predictive accuracy across several tested transcriptomes, suggesting its potential for broader application across different biological systems.
5. To enhance usability, we have developed an online prediction platform. This web server features an intuitive and efficient interface, enabling users to readily submit RNA sequences and obtain m5C site predictions. Consequently, FusDRM-m5C can be more conveniently applied in biological research and various other fields.

Taken together, these contributions highlight the strength of FusDRM-m5C in accurately and robustly identifying m5C sites across species and data conditions. By effectively combining diverse biological features and advanced neural components, our framework provides a reliable tool for predicting transcriptome-wide m5C sites and offers new opportunities for understanding the regulatory roles of RNA methylation in various organisms.

## 2 Materials and methods

### 2.1 Benchmark dataset

For robust model evaluation and fair comparison, we utilized the publicly available benchmark dataset developed by Hasan et al. for their Deepm5C predictor (Hasan et al., 2022). This dataset provides a large-scale, standardized resource for identifying human m5C sites. The construction of this benchmark began with sourcing experimentally validated positive m5C sequences from the m6A-Atlas database (Tang et al., 2021), which initially yielded 95,390 sequences. To minimize redundancy and potential bias, these sequences were processed with CD-HIT (Fu et al., 2012) at a 90% identity threshold, resulting in a final set of 58,159 non-redundant positive samples. Due to the scarcity of experimentally verified non-m5C sites, the negative samples were constructed following a strategy widely adopted in previous studies (Manavalan et al., 2020; Xu et al., 2020; Hasan et al., 2021). Specifically, 41 nt fragments centered on a cytosine were generated from all human chromosomes, and any fragments overlapping with the known positive m5C sites were excluded. This pool was then filtered using CD-HIT (90% threshold) to remove internal redundancy. From this large set of negative candidates, 58,159 sequences were randomly selected to create a balanced dataset with an equal number of positive samples. The rationale for this balanced approach was to prevent the model from developing a bias towards a majority class during training, ensuring that the classifier could effectively learn the distinguishing features of both true m5C sites and non-sites, thereby guaranteeing the reliability and fairness of performance metrics.

All sequences in the dataset are 41 nt long, centered on the cytosine. Crucially, the data was pre-partitioned into distinct training and independent test sets, with 80% of the data serving as the training set and the remaining 20% constituting the independent test set. We adopted this established split, where the training set is used solely for model development and the independent test set is reserved strictly for final performance evaluation. The exact number of positive and negative samples in the training and independent test sets are detailed in Table 1. Using this well-curated, non-redundant, and pre-partitioned benchmark ensures the reliability and comparability of our findings with state-of-the-art methods, which are evaluated on the same data foundation.

**TABLE 1:** Distribution of the benchmark data set.

Dataset component	Positive samples	Negative samples	Total samples
Training set	46,529	46,529	93,038
Independent test set	11,630	11,630	23,260

### 2.2 Feature coding schemes

#### 2.2.1 One-hot encoding

One-hot encoding constitutes a widely accepted technique for translating categorical data, such as nucleotides within an RNA sequence, into a numerical format amenable to machine learning algorithms. This approach transforms each symbolic category into a distinct binary vector. For the four canonical RNA bases $\left\{A,U,G,C\right\}$ , the conventional one-hot mapping is delineated as shown in Equation 1.

$$\begin{array}{c} A\to \left(1,0,0,0\right)\\ U\to \left(0,1,0,0\right)\\ G\to \left(0,0,1,0\right)\\ C\to \left(0,0,0,1\right) \end{array}$$ (1)

In this representation, each nucleotide corresponds to a 4-dimensional vector where only the dimension associated with that specific nucleotide is “hot” (set to 1), while all other dimensions are “cold” (set to 0). Applying this transformation position-by-position along an RNA sequence of length L yields a feature matrix of dimensions 4×L . The primary advantages of one-hot encoding are its simplicity and computational efficiency. The mapping is straightforward and typically implemented via a fast lookup operation. Furthermore, the method generates a clear, orthogonal representation for each nucleotide, thereby precluding the implication of any artificial ordinal relationships. This characteristic proves vital for maintaining the inherent categorical distinctiveness of the bases throughout subsequent analyses.

#### 2.2.2 Z-curve encoding

To capture the sequence composition information, we employed the Z-curve (Zhang and Zhang, 1994; Gao and Zhang, 2004) encoding method. The Z-curve technique provides a unique numerical representation of a DNA or RNA sequence by mapping it into a three-dimensional space, based on the frequency distribution of its constituent bases. The coordinates $\left(x,y,z\right)$ of the curve at a given position n along the sequence reflect the cumulative counts of nucleotides up to that point, as calculated in Equation 2.

$$\left\{\begin{array}{c} x_{n}=\left(A_{n}+G_{n}\right)-\left(C_{n}+U_{n}\right)\\ y_{n}=\left(A_{n}+C_{n}\right)-\left(G_{n}+U_{n}\right)\\ z_{n}=\left(G_{n}+C_{n}\right)-\left(A_{n}+U_{n}\right) \end{array}\right.$$ (2)

For an input RNA sequence of length L , this Z-curve encoding process generates a sequence of L corresponding 3-dimensional vectors. Therefore, the final numerical representation of the sequence used as input for the subsequent model components is a matrix or tensor with the shape L×3 . Each row corresponds to a nucleotide position in the sequence, and the three columns represent the calculated x , y , and z coordinates derived from the cumulative base distribution.

#### 2.2.3 RNA secondary structure encoding

RNA secondary structure (RSS), describing intramolecular base-pairing, provides crucial information beyond the primary sequence. Incorporating this structural information markedly boosts the predictive capabilities of deep learning models across a spectrum of RNA-related bioinformatics applications. Indeed, the efficacy of RNA secondary structure features, frequently represented as graphs, has been widely substantiated in diverse contexts (Zhou et al., 2023; Bai et al., 2025). In this study, we predicted the secondary structure for each RNA sequence using the ViennaRNA Package 2.0 (Lorenz et al., 2011). This package outputs the predicted structure in dot-bracket notation, where parentheses indicate paired bases and dots represent unpaired ones. To make this structural information compatible with our deep learning model, we transformed the dot-bracket string for each RNA into an N × N adjacency matrix, with N being the RNA sequence length. In this matrix, an entry $A_{ij}=1$ signifies a base pair between nucleotides i and j , while $A_{ij}=0$ otherwise. This adjacency matrix, effectively a graph representation of the secondary structure, was then utilized as an input channel for our deep learning model, complementing the primary sequence information.

### 2.3 Model architecture

**FIGURE 1:**
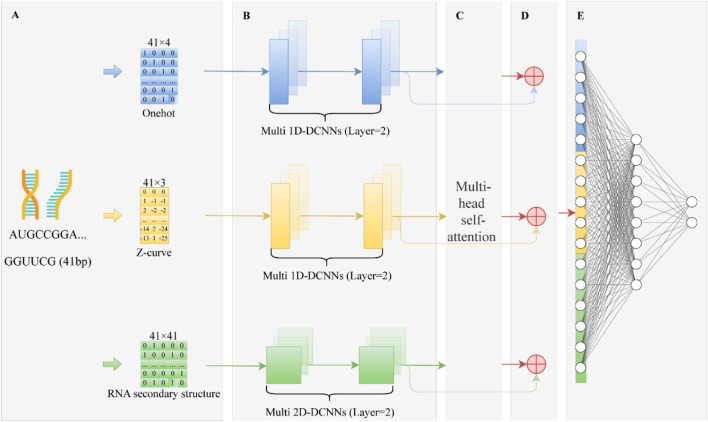
The schematic diagram of FusDRM-m5C. **(A)** Input Features. RNA sequences are encoded using One-hot (41 × 4), Z-curve (41 × 3), and RNA secondary structure (41 × 41). **(B)** Feature Extraction. Each feature type is processed by two-layer dilated CNNs (1D or 2D) to capture both local and long-range patterns. **(C)** Multi-Head Self-Attention (MHSA). Enhances global dependencies across different feature types. **(D)** Residual Fusion. The attention-enhanced features are added to the original CNN outputs via residual connections. **(E)** Classification. A fully connected neural network predicts RNA modification sites based on fused features.

Deep learning has proven to be highly effective in RNA modification site prediction (Huang et al., 2024; Li et al., 2025; Lu et al., 2025), owing to its powerful capability to capture complex patterns and hierarchical features from biological sequences. Building on this foundation, we propose FusDRM-m5C, a deep learning framework designed to accurately predict m5C modification sites by integrating multiple sources of biological information. As shown in Figure 1, FusDRM-m5C employs a multi-branch architecture that processes three complementary types of input features: one-hot encoded nucleotide sequences, Z-curve representations reflecting physicochemical and geometrical properties, and RNA secondary structure matrices capturing local structural context. Each of these feature types is first transformed into a fixed-size matrix representation and then passed through a dedicated convolutional module. Specifically, one-hot and Z-curve features are processed using two layers of 1D Dilated Convolutional Neural Networks (1D-DCNNs), which are effective in extracting multi-scale sequential patterns and long-range dependencies. The secondary structure information, inherently two-dimensional, is handled by a two-layer 2D-DCNN to model spatial interactions between base pairs. After the initial feature extraction stage, the output of each of the three branches is passed through its own multi-head self-attention mechanism. This enables the model to adaptively assign weights and focus on the most informative signals within each feature space. The three resulting feature representations are then integrated through a concatenation operation. Finally, this fused vector is passed to a fully connected layer to produce the ultimate prediction probability of m5C site presence. To improve training stability and promote gradient flow, residual connections are also employed throughout the model.

#### 2.3.1 Dilated convolutional networks

Dilated Convolutions (Yu and Koltun, 2015), also known as Atrous Convolutions, are an effective Convolutional Neural Network (CNN) operation designed to exponentially increase the receptive field of neurons without significantly increasing the number of parameters or computational cost. This is achieved by introducing a fixed gap—the dilation rate ( r )—between the elements of the convolution kernel. When the dilation rate is 1, a dilated convolution is equivalent to a standard convolution. For a 1D input sequence *x* and a filter *w* of size K , the output $y\left[s\right]$ of a dilated convolution at position *s* is given by Equation 3:

$$y\left[s\right]=\sum_{k=0}^{K-1}x\left[s+r\cdot k\right]\cdot w\left[k\right]$$ (3)

Where r is the dilation rate. When r>1 , the kernel skips r−1 elements when processing the input sequence. This effectively enlarges the input region that each output unit “sees” without sacrificing spatial resolution or substantially increasing the number of parameters. In sequence information extraction tasks, especially when processing biological sequences such as RNA or DNA, dilated convolutions enable the capture of longer-range contextual dependencies. By incorporating stacked dilated convolutional layers with diverse (often exponentially increasing) dilation rates, a model is able to establish a hierarchical feature representation. This methodology proficiently integrates sequence information from various scales, proving indispensable for detecting distant conserved patterns or structural features within sequences. Such capability makes dilated convolutions a powerful tool for learning complex patterns from long sequences.

#### 2.3.2 Multi-head self-attention layer

In the field of RNA sequence analysis, particularly for the precise prediction of post-transcriptional RNA modification sites, the Multi-Head Self-Attention (MHSA) (Vaswani et al., 2017) mechanism has become a key technology for constructing deep learning models. This mechanism was originally proposed by Vaswani et al. within the Transformer architecture. For RNA sequences, typically composed of nucleotides, MHSA can effectively capture complex long-range dependencies and local contextual features within the sequence, which is instrumental in identifying key sequence patterns that determine specific RNA modifications. When an input RNA sequence is processed and converted into a numerical representation X via an embedding layer, MHSA projects X into h different representation subspaces, enabling the model to scrutinize sequence information from multiple perspectives in parallel. For the i -th attention head ( i=1,…,h ), its specific query $Q_{i}$ , key $K_{i}$ , and value $V_{i}$ are obtained through the mappings given in Equation 4:

$$\begin{array}{c} Q_{i}=XW_{i}^{Q}\\ K_{i}=XW_{i}^{K}\\ V_{i}=XW_{i}^{V} \end{array}$$ (4)

Here, $W_{i}^{Q}\in R^{d_{embed}\times d_{k}}$ , $W_{i}^{K}\in R^{d_{embed}\times d_{k}}$ , and $W_{i}^{V}\in R^{d_{embed}\times d_{v}}$ are learnable projection parameter matrices, $d_{embed}$ is the nucleotide embedding dimension, and $d_{k}$ and $d_{v}$ are the key and value dimensions, respectively, typically set to $d_{embed}/h$ Each head then independently computes its attention as shown in Equation 5:

$${head}_{i}=Attention\left(Q_{i},K_{i},V_{i}\right)=softmax\left(\frac{Q_{i}K_{i}^{T}}{\sqrt{d_{k}}}\right)V_{i}$$ (5)

The scaling factor $\sqrt{d_{k}}$ herein helps stabilize gradients. In this manner, different heads might learn to focus on distinct features within the RNA sequence, such as specific conserved motifs, nucleotides at particular distances from the target modification site, or patterns indicative of local secondary structures. The outputs of these parallelly computed attention heads are then concatenated and passed through a final linear transformation to obtain the aggregated output of the MHSA layer, as shown in Equation 6:

$$MultiHead\left(X\right)=Concat\left({head}_{i},\ldots ,{head}_{i}\right)W^{O}$$ (6)

where $W^{O}\in R^{hd_{v}\times d_{model}}$ is the output projection matrix, and $d_{model}$ is often consistent with the input embedding dimension $d_{embed}$ . In the context of RNA modification site prediction, the contextual representations learned by MHSA enable the model to integrate signals from different regions of the RNA sequence that contribute to the classification decision, thereby enhancing its ability to distinguish true modification sites from unmodified ones.

#### 2.3.3 Residual connection

Residual Connections are a groundbreaking technique in deep learning, first introduced by He et al. in their Residual Networks (ResNets) (He et al., 2016), designed to address the degradation problem encountered when training very deep neural networks. As network depth increases, model performance often saturates and then degrades rapidly, a phenomenon not entirely caused by overfitting but rather by the difficulty of optimizing deep architectures. Residual connections alleviate this issue by introducing “shortcut connections” that allow information to bypass one or more layers. Specifically, if the desired underlying mapping to be learned by a few stacked layers is $H\left(X\right)$ , the residual learning framework lets these layers fit a residual mapping $F\left(X\right)=H\left(X\right)-x$ . The original mapping is thus recast as $F\left(X\right)+x$ . The output *y* of a residual block is defined by Equation 7:

$$y=F\left(x,\left\{W_{i}\right\}\right)+x$$ (7)

where x*x* is the input, and $F\left(x,\left\{W_{i}\right\}\right)$ represents the residual function learned by the layers with weights $\left\{W_{i}\right\}$ . This formulation enables layers to learn an identity mapping easily. If $F\left(x,\left\{W_{i}\right\}\right)$ contributes little, thereby not impeding effective information flow. More importantly, residual connections significantly improve gradient propagation during backpropagation, enabling the effective training of substantially deeper networks. This has led to significant performance gains in various computer vision tasks and other domains, including sequence modeling. Residual connections have since become a standard component in the design of deep neural networks.

#### 2.3.4 Fully connected neural network

Fully Connected Neural Networks (FCNNs), often referred to as Dense Layers or Multilayer Perceptrons (MLPs), represent a foundational architecture in neural networks where each neuron in one layer is connected to every neuron in the subsequent layer. This dense connectivity allows FCNNs to learn complex, non-linear relationships between inputs and outputs. The computation within a single fully connected layer l transforming an input vector $h^{\left(l-1\right)}$ to an output vector $h^{\left(l\right)}$ is given by Equation 8.

$$h^{\left(l\right)}=\sigma \left(W^{\left(l\right)}h^{\left(l-1\right)}+b^{\left(l\right)}\right)$$ (8)

where $W^{\left(l\right)}$ is the weight matrix, $b^{\left(l\right)}$ is the bias vector for layer l , and σ denotes a non-linear activation function, such as the Rectified Linear Unit (ReLU), sigmoid, or tanh function. Through the stacking of multiple such layers, FCNNs can approximate a wide range of functions, making them powerful universal function approximators. While Convolutional Neural Networks (CNNs) or Recurrent Neural Networks (RNNs) might be more efficient for data with specific structures, FCNNs remain crucial components in many deep learning models, often employed as classification or regression modules following feature extraction, or as key transformation layers within more complex architectures.

### 2.4 Performance evaluation

To comprehensively assess the predictive capability of the proposed FusDRM-m5C model, its performance was evaluated using five widely recognized statistical metrics: Sensitivity (SN), Specificity (SP), Accuracy (ACC), Matthews correlation coefficient (MCC), and the Area Under the Receiver Operating Characteristic Curve (AUC). The formulas used for calculation are given in Equation 9:

$$\left\{\begin{array}{l} Sn=\frac{TP}{TP+TN}\\ Sp=\frac{TN}{TN+FP}\\ Acc=\frac{TP+TN}{TP+TN+FP+FN}\\ Mcc=\frac{TP\times TN-FP\times FN}{\sqrt{\left(TP+FP\right)\times \left(TP+FN\right)\times \left(TN+FP\right)\times \left(TN+FN\right)}}) \end{array}\right.$$ (9)

The terms True Positives (TP), False Positives (FP), True Negatives (TN), and False Negatives (FN) used in the formulas above represent counts derived from the classification results. Specifically, TP is the number of actual positive instances correctly identified as positive; FP is the number of negative instances incorrectly classified as positive; TN is the number of actual negative instances correctly identified as negative; and FN is the number of actual positive instances incorrectly classified as negative. The Area Under the Receiver Operating Characteristic Curve (AUC), calculated based on the ROC curve, further summarizes the model’s overall ability to discriminate between the positive and negative classes across all possible classification thresholds, with a value closer to 1 indicating better discriminatory power. While all these metrics (SN, SP, ACC, MCC, AUC) offer valuable insights, in this study, we placed particular emphasis on ACC and MCC as key indicators reflecting the model’s overall predictive accuracy and its balanced performance across both classes.

### 2.5 Instructions for setting hyperparameters

To ensure a fair comparison with existing studies, the FusDRM-m5C model was trained on the identical dataset. Computational acceleration was facilitated by an NVIDIA GeForce RTX 4080 GPU. During training, the Adam with Weight Decay (AdamW) optimizer was employed to enhance the stability of gradient updates and mitigate convergence to local optima. To prevent model overfitting, a combination of strategies including regularization, dropout, and early stopping was utilized. Hyperparameters were optimized via comparative experiments: the learning rate was fixed at $1\times e^{-4}$ after logarithmic search in $1\times e^{-5}-1\times e^{-2}$ ; the batch size was set to 64 considering both efficiency and generalization; and a dropout rate of 0.2 was chosen to reduce overfitting. The maximum number of epochs was limited to 300, with early stopping triggered if validation loss failed to improve for 30 consecutive epochs. Network depth was determined through ablation experiments to balance representational capacity and model complexity. The implementation was conducted in Python 3.10 with PyTorch 2.0.0 + cu11.8, and the final hyperparameter settings are summarized in Table 2.

**TABLE 2:** Description of the hyperparameters in the FusDRM-m5C model.

Parameters	Number
Layer 1 dilation rate	1
Layer 1 kernel size	3
Layer 2 dilation rate	2
Layer 2 kernel size	5
Num heads	4
Drop out	0.2
FCNNs layer sizes	[128, 64, 32, 16, 8, 4, 2]

## 3 Result and discussion

### 3.1 Classification performance and computational efficiency

**FIGURE 2:**
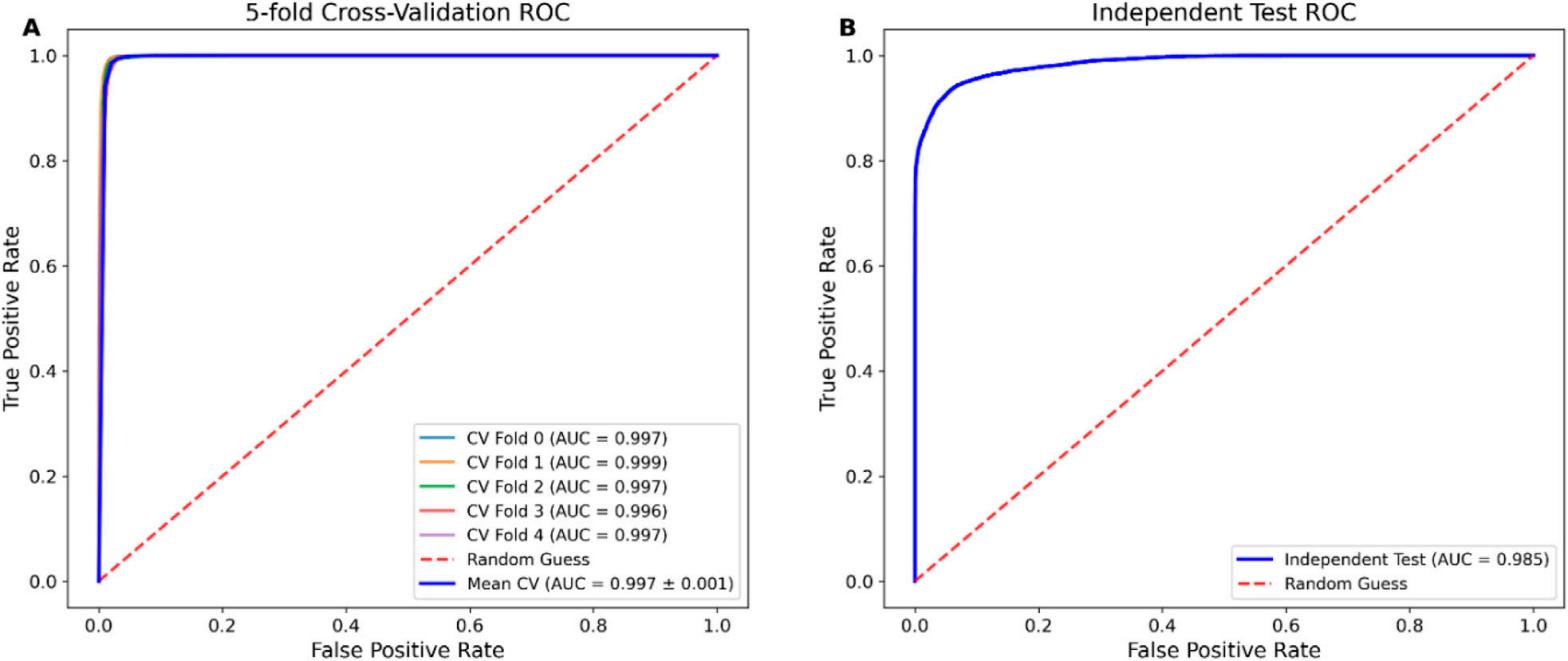
**(A)** ROC curves of 5-fold cross-validation. **(B)** ROC curves of the independent test.

To comprehensively evaluate the classification performance of our proposed FusDRM-m5C model, we conducted both 5-fold cross-validation and independent testing on the benchmark dataset. During 5-fold cross-validation, the model demonstrated consistently high predictive accuracy across all folds, with sensitivity, specificity, accuracy, Matthews correlation coefficient (MCC), and area under the ROC curve (AUC) reaching 0.992, 0.971, 0.982, 0.946, and 0.997, respectively. The receiver operating characteristic (ROC) curves from each fold tightly converged near the top-left corner, clearly indicating minimal variability and robust generalization within the training data. Of particular note, the average AUC across all folds reached 0.994, accompanied by a low standard deviation of merely ±0.001, further solidifying the model’s overall robustness (Figure 2A). Evaluation on the independent test set revealed a slight yet acceptable drop in performance, with sensitivity, specificity, accuracy, MCC, and AUC values of 0.900, 0.965, 0.932, 0.867, and 0.985, respectively. Despite this reduction, the high AUC and balanced Sn and Sp demonstrate that the model maintains strong discriminative capability on unseen data. Furthermore, the ROC curve generated from the independent test set exhibits a steep upward trend, effectively distinguishing positive from negative instances (Figure 2B). Collectively, these findings indicate that the model possesses strong classification capability and promising generalization performance, highlighting its potential practical utility for m5C site prediction in both in-sample and out-of-sample contexts.

In addition to its classification performance, the model’s computational efficiency was tested on a computing platform running Windows 11 with an AMD Ryzen 9 7840HX processor, 16.0 GB of RAM, and an NVIDIA GeForce RTX 4060 GPU. As detailed in Table 3, the model demonstrates significant acceleration capabilities when utilizing a GPU, achieving a speed-up of approximately 4.6 times compared to using only the CPU for 50,000 samples. It should also be noted that the model requires an initial overhead of about 38 s for import and warm-up on its first run; this one-time cost is not included in the net prediction times in the table, and its impact is amortized when processing large-scale or continuous tasks. While the GPU acceleration is significant, the model’s value is further highlighted by its broad accessibility. Even in a CPU-only environment, its performance remains highly efficient—processing a task of 50,000 samples in approximately 19 s. This result indicates that the model’s performance in a CPU environment also makes it practically usable for researchers with limited computational resources, which helps to broaden its scope of application as a research tool.

**TABLE 3:** Computational performance of the FusDRM-m5C model with varying sample sizes.

Sample size	CPU only	GPU used
2000	00:00.860	00:00.196
10,000	00:04.094	00:00.893
50,000	00:19.323	00:04.228

Time format is presented as minutes:seconds.milliseconds.

### 3.2 Comparison with CNN variants

To systematically evaluate the performance of different convolutional neural network variants, we trained the standard CNN, Dilated Convolutional Neural Network (DCNN), Temporal Convolutional Network (TCN) (Bai et al., 2018), and Densely Connected Convolutional Networks (DenseNet) (Huang et al., 2017) on the same dataset. The average metrics were obtained through 5-fold cross-validation, with the results presented in Table 4, where all metrics are reported as mean ± standard deviation. The standard CNN showed relatively limited performance, with an accuracy of 0.808 ± 0.036, suggesting that its shallow architecture is insufficient for modeling complex sequential features. DenseNet, while effective in enhancing feature reuse through dense connections in image-related tasks, achieved an accuracy of only 0.833 ± 0.044 in this study, indicating a limited capacity for capturing long-range dependencies within sequences. In contrast, TCN, which integrates causal and dilated convolutions, demonstrated a strong ability to capture temporal dependencies, achieving a higher accuracy of 0.952 ± 0.020. The DCNN achieved the best performance across all metrics, with an accuracy of 0.983 ± 0.003, an MCC of 0.966 ± 0.006, and an AUC of 0.997 ± 0.001. The use of dilated convolutions significantly enlarges the receptive field without increasing the number of parameters, enabling the model to simultaneously capture both local details and global contextual information. This property explains why DCNN exhibited superior feature representation and classification performance compared with the other CNN variants.

**TABLE 4:** Performance comparison of CNN and its variants under 5-fold cross-validation.

Method	Sn	Sp	ACC	MCC	AUC
CNN	0.808 ± 0.044	0.809 ± 0.034	0.808 ± 0.036	0.616 ± 0.072	0.882 ± 0.035
DCNN	**0.995** ± **0.003**	**0.971** ± **0.005**	**0.983** ± **0.003**	**0.966** ± **0.006**	**0.997** ± **0.001**
TCN	0.943 ± 0.032	0.961 ± 0.011	0.952 ± 0.020	0.905 ± 0.040	0.988 ± 0.007
Densenet	0.772 ± 0.055	0.895 ± 0.037	0.833 ± 0.044	0.672 ± 0.086	0.908 ± 0.040

Each value in the table is expressed as mean ± SD. where the mean is the average of the 5-fold cross-validation results and SD is their standard deviation. The best experimental results are highlighted in bold.

In summary, although TCN and DenseNet achieved competitive performance in certain metrics, the dilated convolution-based DCNN consistently demonstrated the best and most stable results in the 5-fold cross-validation. Therefore, DCNN was selected as the core model in this study to better capture sequential features and enhance prediction accuracy.

### 3.3 Ablation study

**FIGURE 3:**
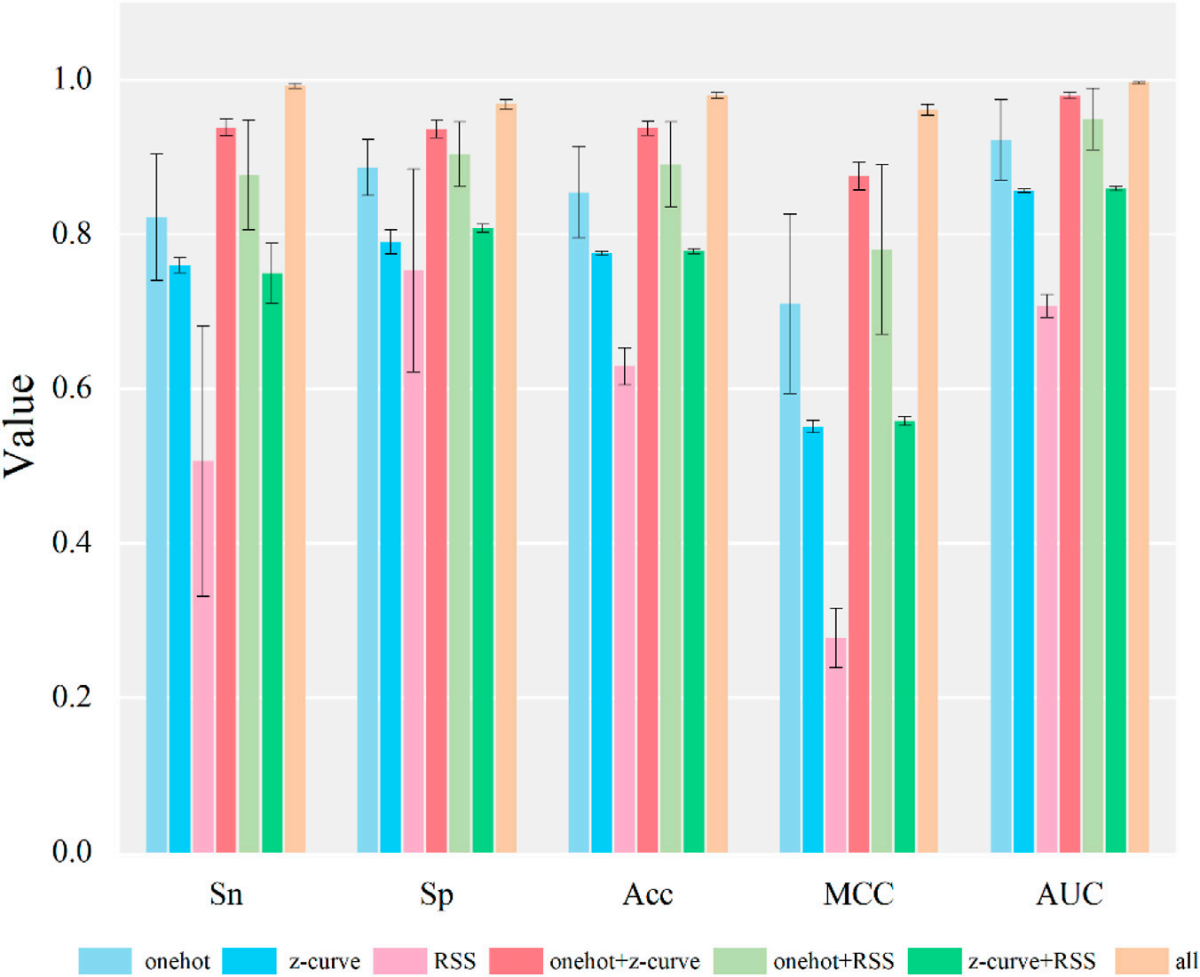
Performance comparison of different feature encoding strategies. This figure shows the classification performance of different single and combined feature encoding methods evaluated using five metrics—Sensitivity (Sn), Specificity (Sp), Accuracy (ACC), Matthews correlation coefficient (MCC) and Area Under the ROC Curve (AUC). The fused representation (all) achieves the highest performance across all metrics. Error bars indicate the standard deviation.

To elucidate the contribution of individual feature encoding schemes and architectural components to the overall predictive performance, comprehensive ablation studies were conducted. To assess the efficacy of various feature encoding strategies and their synergistic effects, a systematic investigation was conducted, as detailed in Figure 3. Among the standalone feature sets, “one-hot” encoding established a solid baseline, particularly excelling in sensitivity (Sn) and accuracy (ACC). In contrast, “z-curve” and “RSS” features, when considered individually, presented varied outcomes. Specifically, “RSS” consistently registered inferior scores across the majority of metrics, most notably for MCC. Crucially, the integration of features in pairwise combinations, such as “one-hot+z-curve” and “one-hot+RSS,” frequently led to substantial performance gains compared to their isolated counterparts, thereby affirming their inherent complementarity. However, the most significant and consistent enhancement across all evaluated metrics—Sn, specificity (Sp), ACC, Matthews correlation coefficient (MCC), and Area Under the Curve (AUC)—was achieved when all feature types were integrated (‘all’). These findings underscore the synergistic advantages offered by a holistic feature representation. By integrating diverse facets of the sequence information, such a representation significantly boosts predictive accuracy, ultimately yielding the highest AUC and a well-rounded overall performance.

**FIGURE 4:**
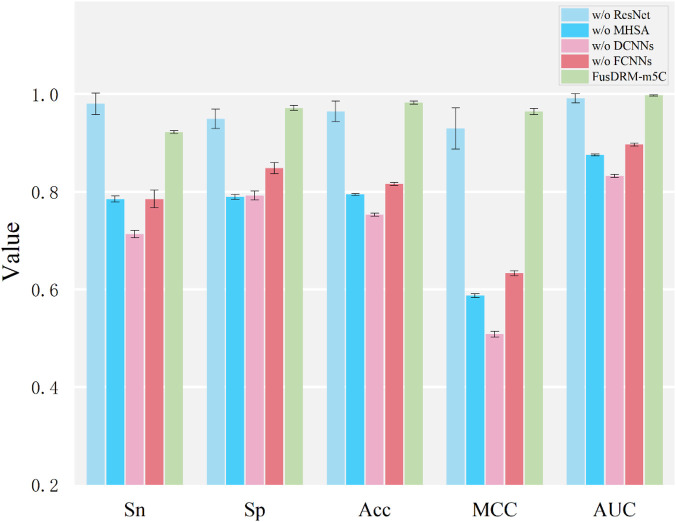
Ablation study on FusDRM-m5C model architecture. This figure shows the predictive performance of FusDRM-m5C when individual components are removed. Evaluation metrics include Sensitivity (Sn), Specificity (Sp), Accuracy (ACC), Matthews correlation coefficient (MCC), and Area Under the Curve (AUC). The term *w/o* means the model is tested without the corresponding module. FusDRM-m5C refers to the complete model. Error bars indicate the standard deviation.

Similarly, Figure 4 presents the results of ablating key architectural components of the proposed FusDRM-m5C model to assess their individual importance. The removal of each component—ResNet (“w/o ResNet”), Multi-Head Self-Attention (“w/o MHSA”), Deep Convolutional Neural Networks (“w/o DCNNs”), and Fully Connected Neural Networks (“w/o FCNNs”)—resulted in a discernible degradation of performance compared to the complete FusDRM-m5C model. The omission of DCNNs resulted in a significant decline across all evaluated metrics, with MCC and AUC showing the most pronounced deterioration. This underscores their critical contribution to extracting essential hierarchical features. Similarly, the absence of MHSA also visibly diminished performance, highlighting its effectiveness in capturing long-range dependencies. While the removal of ResNet and FCNNs also diminished predictive capabilities, the complete FusDRM-m5C model, which integrates all these components, consistently achieved the highest scores across Sn, Sp, ACC, MCC, and AUC. This affirms that each architectural element contributes uniquely and synergistically to the model’s overall efficacy, justifying the integrated design of FusDRM-m5C.

### 3.4 Visualization with T-distributed stochastic neighbor embedding

**FIGURE 5:**
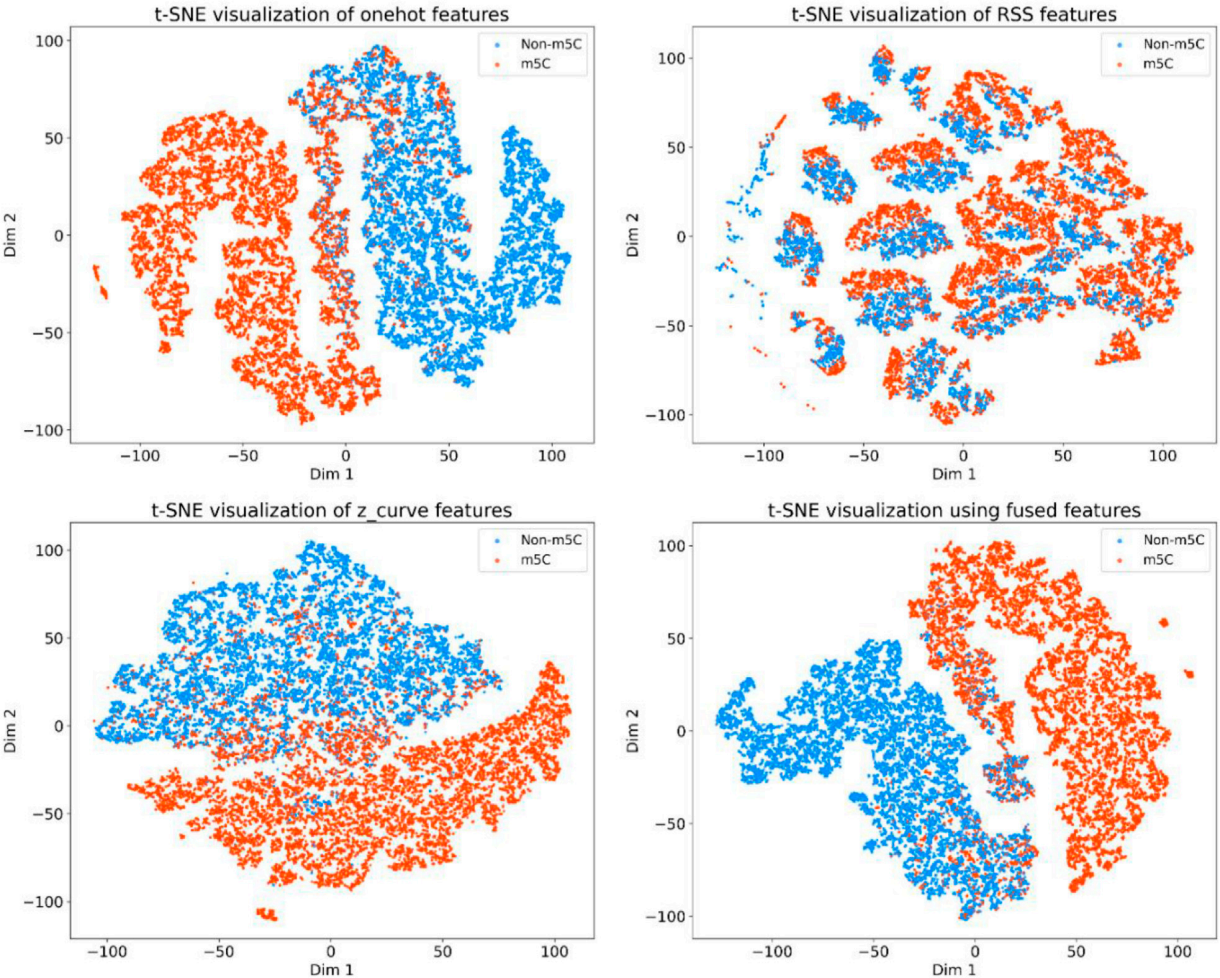
t-SNE visualization of features from the test set.

To qualitatively assess the discriminative capabilities of different feature encoding strategies and the benefit of their integration, t-SNE (t-distributed Stochastic Neighbor Embedding) was employed to visualize the high-dimensional feature spaces in two dimensions (Figure 5). The t-SNE plot for one-hot features reveals that Non-m5C (blue) and m5C (orange) samples form two relatively distinct, albeit somewhat elongated and intermingled, primary clusters. This suggests that one-hot encoding provides a foundational level of separability. In stark contrast, the visualization for RSS (RNA Secondary Structure) features shows a highly fragmented distribution. Both Non-m5C and m5C samples are scattered across numerous small, overlapping sub-clusters, indicating that RSS features alone offer limited discriminative power for these classes. The z-curve features present an intermediate scenario. While some clustering is evident, with a general separation between a larger blue mass and a more dispersed orange region, there is still considerable overlap, particularly at the interface between the two classes. Crucially, the t-SNE visualization of the testing set, which implicitly utilizes the fused representation of all three feature types, demonstrates a marked improvement in class separability. The Non-m5C and m5C samples form more coherent, compact, and distinctly segregated clusters with a visibly clearer boundary between them compared to any of the individual feature sets. This visual evidence strongly supports the hypothesis that the fusion of diverse feature encodings (one-hot, z-curve, and RSS) captures a more comprehensive and discriminative representation, thereby enhancing the model’s ability to distinguish between m5C and Non-m5C sites.

### 3.5 Performance comparison with existing methods

To better understand the strengths and limitations of different m5C predictors under consistent evaluation settings, we compared FusDRM-m5C with four representative models: Deepm5C (Hasan et al., 2022), im5C-DSCGA (Jia et al., 2023), m5C-Seq (Abbas et al., 2024), and im7G-DCT (Lei et al., 2025). All methods were retrained and tested on the same datasets to ensure fair and objective comparison. As summarized in Table 5, FusDRM-m5C achieved the best overall performance in 5-fold cross-validation. Its sensitivity was 0.995, specificity was 0.971, accuracy reached 0.983, MCC was 0.966, and AUC was 0.997, all surpassing those of existing methods. Among the baselines, im7G-DCT performed the strongest, yet FusDRM-m5C outperformed it by 32.33% in MCC and 6.29% in AUC. These metrics are particularly important in imbalanced classification tasks. AUC gauges a model’s effectiveness in distinguishing between classes across various thresholds. MCC, on the other hand, offers a more robust measure of prediction quality by integrating all four components of the confusion matrix. The substantial improvements suggest that FusDRM-m5C not only excels in class separation but also produces balanced and reliable outputs across both positive and negative instances.

**TABLE 5:** Comparison of FusDRM-m5C with existing models.

Method	Sn	Sp	ACC	MCC	AUC
Deepm5C	0.835 ± 0.06	0.875 ± 0.01	0.855 ± 0.07	0.697 ± 0.10	0.941 ± 0.05
im5C-DSCGA	0.814 ± 0.02	0.885 ± 0.02	0.850 ± 0.00	0.721 ± 0.00	0.926 ± 0.00
m5C-seq	0.821 ± 0.001	0.862 ± 0.004	0.841 ± 0.001	0.683 ± 0.002	0.888 ± 0.002
im7G-DCT	0.851 ± 0.04	0.879 ± 0.01	0.865 ± 0.02	0.730 ± 0.04	0.938 ± 0.02
FusDRM-m5C	**0.995** ± **0.003**	**0.971** ± **0.005**	**0.983** ± **0.003**	**0.966** ± **0.006**	**0.997** ± **0.001**

Each value in the table is expressed as mean ± SD. where the mean is the average of the 5-fold cross-validation results and SD is their standard deviation. The best experimental results are highlighted in bold.

On the independent test set, as shown in Table 6, FusDRM-m5C demonstrated strong generalization ability, achieving an ACC of 0.933 and an MCC of 0.867, both the highest among the evaluated models. Compared to im7G-DCT, it improved MCC by 21.94% and AUC by 6.59%, confirming that the model’s advantage is not limited to the training data. In terms of sensitivity and specificity, FusDRM-m5C achieved a balanced performance with values of 0.900 and 0.965, respectively. In contrast, other models tended to prioritize one over the other. For instance, im5C-DSCGA showed high specificity but lower sensitivity, while Deepm5C displayed the opposite trend. Such balance is critical in biological applications where both false positives and false negatives can have significant downstream consequences.

**TABLE 6:** Comparison of FusDRM-m5C with the existing predictor on independent dataset.

Method	Sn	Sp	ACC	MCC	AUC
Deepm5C	0.846	0.857	0.852	0.691	0.938
im5C-DSCGA	0.810	0.908	0.859	0.721	0.926
m5C-seq	0.852	0.806	0.827	0.657	0.870
im7G-DCT	0.811	0.897	0.854	0.711	0.925
FusDRM-m5C	**0.900**	**0.965**	**0.933**	**0.867**	**0.986**

The best experimental results are highlighted in bold.

Furthermore, an examination of the relative performance among existing methods reveals inherent limitations concerning either their feature extraction strategies or their underlying model architectures. For instance, methods like Deepm5C and m5C-Seq often exhibited only moderate AUC scores and comparatively low MCC values. This suggests a struggle in effectively capturing ambiguous sequence signals or, alternatively, in consistently generating robust predictions. Although im7G-DCT incorporated dual-channel transformations to enhance feature representation, its performance plateaued. This suggests that further modeling of contextual dependencies and hierarchical patterns may be necessary—areas where FusDRM-m5C offers specific advantages.

In summary, these comprehensive results suggest that FusDRM-m5C can achieve a high level of prediction performance with good generalization and a well-balanced output on the tested datasets. These strengths support its potential as a reliable tool for transcriptome-wide identification of m5C sites.

### 3.6 Cross-species performance of the FusDRM-m5C model

The purpose of this analysis was to assess the cross-species generalization performance of the FusDRM-m5C prediction tool. This assessment utilized the benchmark datasets described in the work of Abbas et al. (2024), which were constructed from the m5C-Atlas database (Ma et al., 2022), with the specific distribution detailed in Table 7. These datasets, covering *M. musculus*, *H. sapiens*, *D. melanogaster*, and *D. rerio*, were processed to create balanced positive and negative samples, which were then split into distinct training and independent test sets. The models were trained on one species’ training dataset and evaluated on the independent test sets from all four species.

**TABLE 7:** Distribution of the benchmark data set.

Species	Training			Independent		
	Positive	Negative	All	Positive	Negative	All
*M. musculus*	8,489	8,489	16,978	2,122	2,122	4,244
*H. Sapiens*	65,543	65,543	131,086	16,386	16,386	32,772
*D. melanogaster*	3,010	3,010	6,020	752	752	1,504
*D. rerio*	4,814	4,814	9,628	1,204	1,204	2,408

**FIGURE 6:**
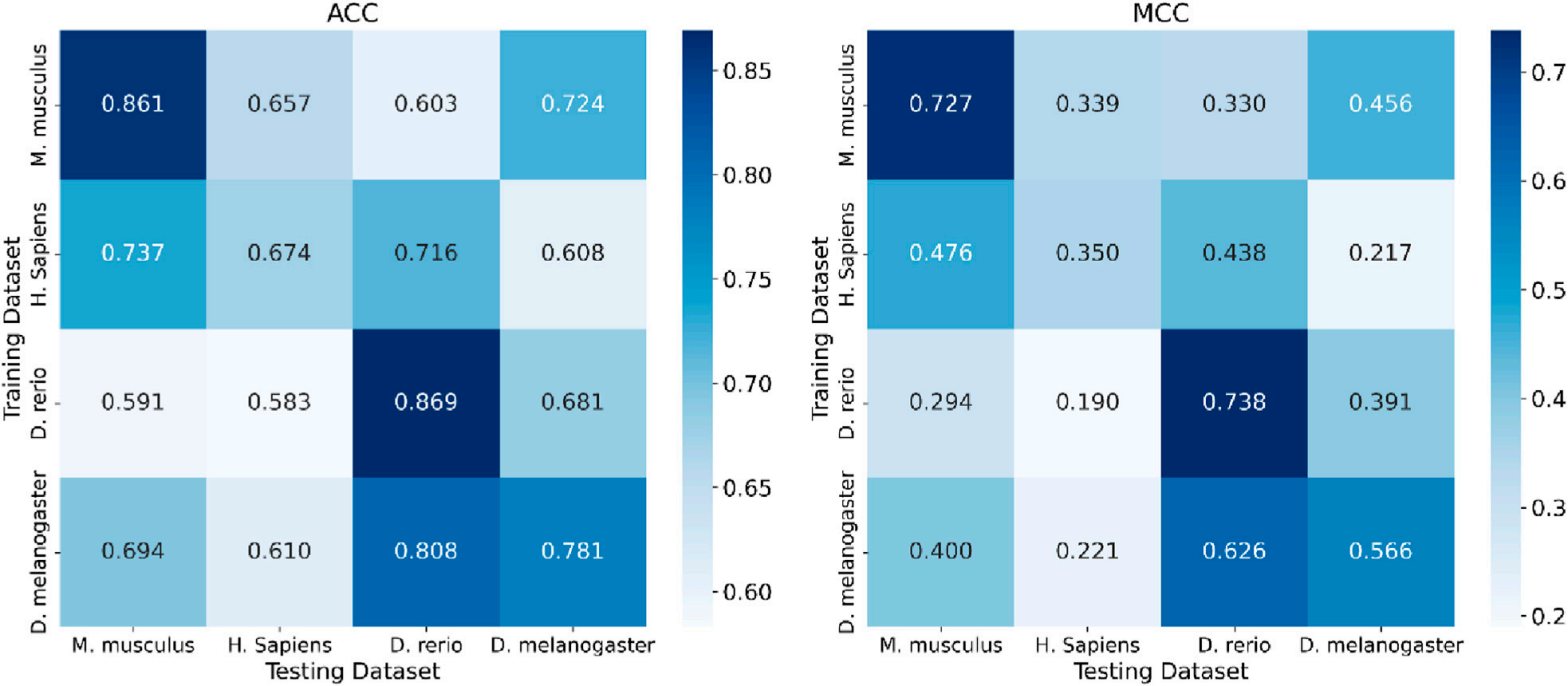
Cross-species generalization performance of a predictive model. Heatmaps display Accuracy (ACC) and Matthews correlation coefficient (MCC) assessing the model’s predictive generalization when trained on one species and tested across different organisms.

Table 8 highlights FusDRM-m5C’s superior intra-species predictive performance over existing methods. The heatmaps (Figure 6) further visualize this, with models trained and tested on the same species, which correspond to the diagonal elements, consistently yielding the highest ACC and MCC scores; for example, these values were 0.861/0.727 for *M. musculus* and 0.869/0.738 for *D. rerio*. Critically, FusDRM-m5C also demonstrates notable cross-species generalization, exemplified by a model trained on *D. melanogaster* achieving a remarkable ACC of 0.808 and an MCC of 0.626 when tested on *D. rerio*; this suggests the capture of deeply conserved m5C regulatory features or highly generalizable sequence patterns. Similarly, training on *H. sapiens* resulted in strong predictive power on *M. musculus*, achieving an ACC of 0.737 and an MCC of 0.476, which demonstrates effective knowledge transfer between these closely related mammals. However, performance varied across other species pairs; training on *D. rerio* and testing on *H. sapiens*, for instance, yielded lower scores with an ACC of 0.583 and an MCC of 0.190, a trend also observed when training on *H. sapiens* and testing on *D. melanogaster*, which produced an ACC of 0.608 and an MCC of 0.217. These observed discrepancies could stem from various factors, including differing evolutionary conservation of m5C motifs, variations in sequence context preferences, or even specific characteristics of the datasets employed. Nevertheless, the overall findings clearly highlight FusDRM-m5C’s capability to learn transferable features. This firmly positions it as a highly promising tool for m5C site prediction, particularly valuable in situations where species-specific data is scarce.

**TABLE 8:** Comparison of FusDRM-m5C with the existing predictor on independent dataset.

Datasets	Method	Sn	Sp	ACC	MCC	AUC
*M. musculus*	m5C-seq	0.713	0.701	0.707	0.413	0.765
	m5CStack	0.722	**0.734**	0.728	0.457	-
	FusDRM-m5C	**0.925**	0.679	**0.861**	**0.727**	**0.940**
*H. sapiens*	m5C-seq	0.624	0.612	0.618	0.236	0.658
	m5CStack	0.622	**0.650**	0.631	0.262	-
	FusDRM-m5C	**0.706**	0.621	**0.674**	**0.350**	**0.741**
*D. rerio*	m5C-seq	0.729	**0.760**	0.743	0.487	0.799
	m5CStack	0.778	0.753	0.765	0.531	-
	FusDRM-m5C	**0.877**	0.719	**0.869**	**0.738**	**0.943**
*D. melanogaster*	m5C-seq	0.761	0.755	0.758	0.516	0.814
	m5CStack	0.749	**0.798**	0.773	0.547	-
	FusDRM-m5C	**0.836**	0.517	**0.781**	**0.566**	**0.858**

The best experimental results are highlighted in bold.

### 3.7 Cross-species comparison of Sequence Motifs

**FIGURE 7:**
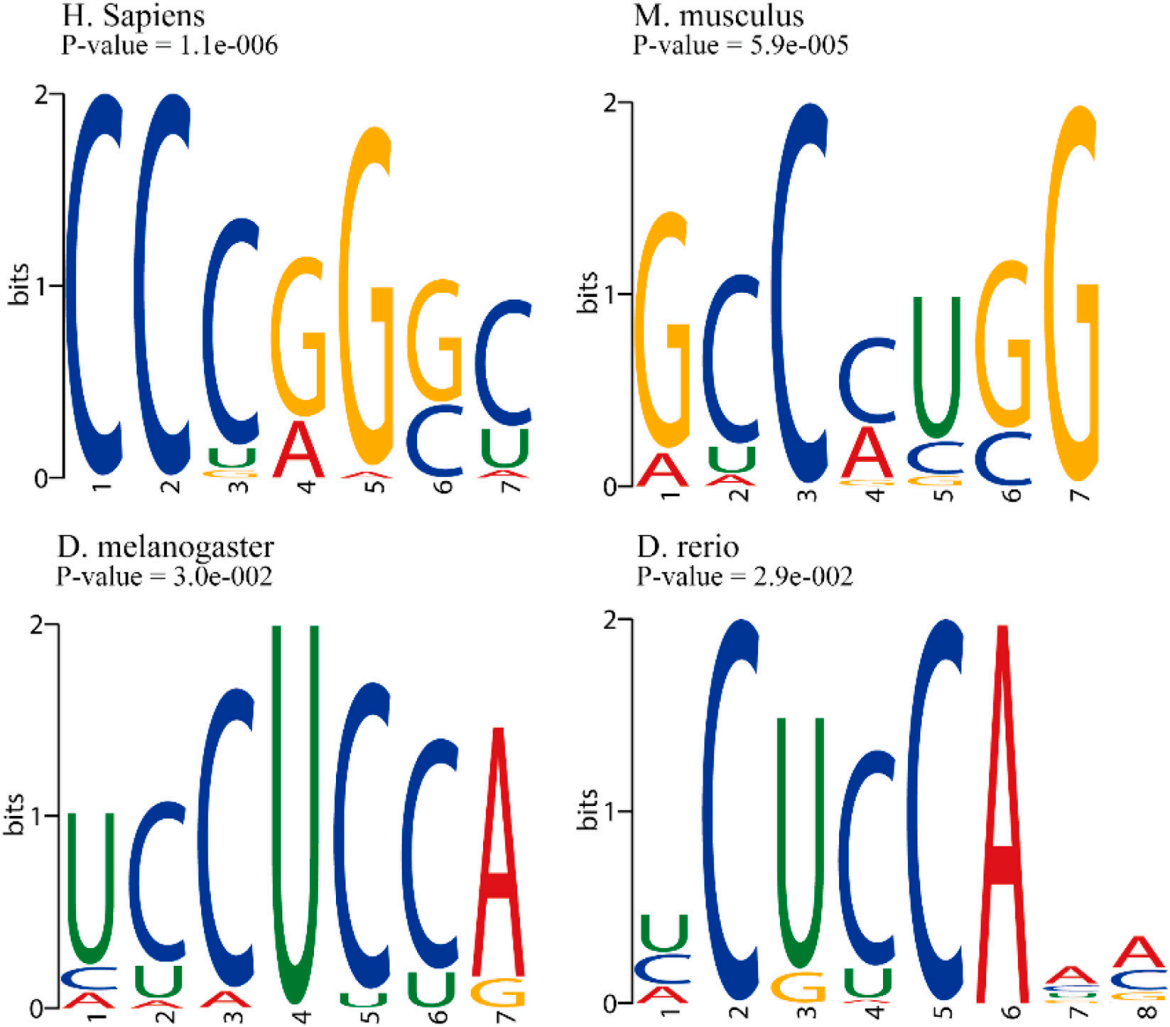
Representative m5C Sequence Motifs. Sequence logos generated by STREME analysis for different species, illustrating both potentially conserved patterns and species-specific sequence preferences surrounding m5C modification sites.

Considering the substantial performance variations we observed, especially the pronounced variability and asymmetry in cross-species predictions, these findings prompted us to conduct a focused cross-species motif analysis. Our goal was to obtain more profound mechanistic insights and to elucidate the sequence features driving these discrepancies. To this end, we employed the STREME (Bailey, 2021) tool from the MEME Suite (Bailey et al., 2009) to perform a comparative motif analysis on sequences surrounding m5C sites across the different species. Figure 7 visually corroborates the potential basis for these performance discrepancies by revealing distinct m5C consensus motifs across the studied species. Notably, mammalian species, *H. sapiens* and *M. musculus*, exhibit predominantly G/C-rich motifs. *H. sapiens* displays a strong “CCCGGGC” consensus, while *M. musculus* shows a related but distinct “GCCCUGG” pattern. In stark contrast, *D. melanogaster* and *D. rerio* present pyrimidine-rich motifs, characterized by “UCCUCCA” and “CUCCA,” respectively. The distinct disparity between the G/C-rich motifs found in mammals and the U/C-rich motifs characteristic of non-mammalian species provides a strong rationale for the observed limited transferability of models across these taxonomic groups.

To more quantitatively assess these motif similarities, we performed a pairwise comparison of the consensus motifs discovered across species using the TOMTOM (Gupta et al., 2007; Tanaka et al., 2011) tool from the MEME Suite. The data presented in Table 9 provides strong statistical support for the performance discrepancies we observed. For instance, the motifs between the two mammalian species, *H. sapiens* and *M. musculus*, are not significantly similar (p = 0.217), which aligns with the asymmetric predictive performance between them. More strikingly, the motifs of the non-mammalian species *D. rerio* and *D. melanogaster* show significant similarity (p = 0.015), explaining the better cross-species prediction performance observed between them. Conversely, comparisons between mammalian and non-mammalian motifs (e.g., *H. sapiens* vs. *D. rerio*, p = 0.927) show high dissimilarity, which is consistent with the observed limited transferability of models across these taxonomic groups. Furthermore, even within these evolutionarily closer species, subtle variations in nucleotide composition and positional preferences—such as differences in G/C abundance or pyrimidine positioning—likely contribute to asymmetric predictive performance. The insights gained from these observations point to the possibility that m5C-associated enzymes likely target highly specific and evolutionarily constrained sequence contexts. These contexts, in turn, appear to be sculpted by both species-specific regulatory demands and prevailing selective pressures. Consequently, prediction models trained on one species may fail to fully capture the discriminative features required for accurate m5C site identification in another, emphasizing the need to account for interspecies variation when developing cross-species epitranscriptomic tools.

**TABLE 9:** Cross-species motif similarity comparison.

Species	*H. sapiens*	*M. musculus*	*D. rerio*	*D. melanogaster*
*H. sapiens*	N/A	0.217	0.927	0.927
*M. musculus*	0.217	N/A	0.658	0.466
*D. rerio*	0.927	0.658	N/A	**0.015**
*D. melanogaster*	0.927	0.466	**0.015**	N/A

Tomtom similarity comparison p-values for m5C motifs across the four species. Lower p-values indicate greater similarity. Bold indicates statistical significance (p < 0.05); N/A denotes self-comparison.

### 3.8 Web server

**FIGURE 8:**
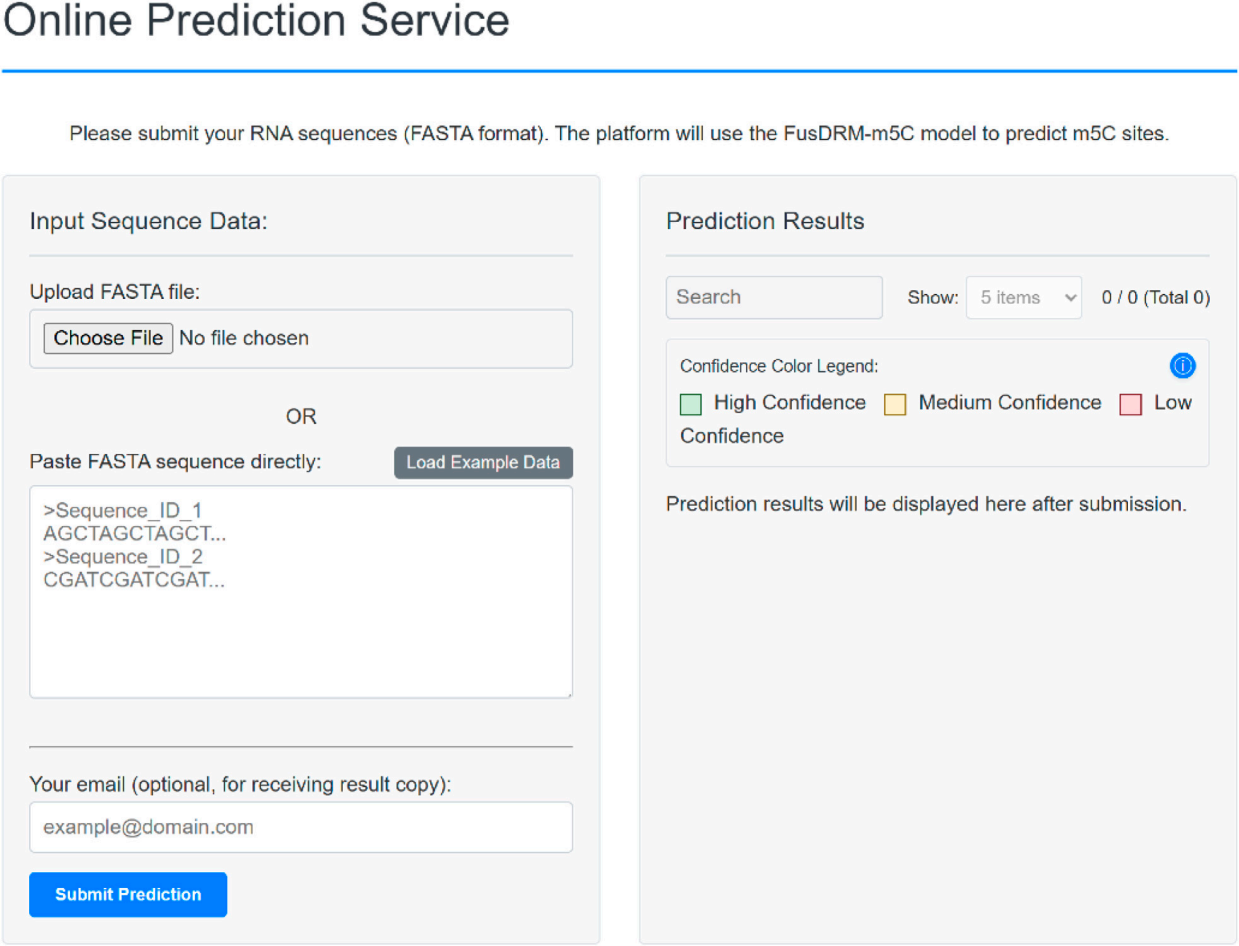
FusDRM-m5C web interface: Input submission and result display modules.

To maximize the utility and accessibility of our FusDRM-m5C model, we have developed and deployed a user-friendly web server, freely available at https://complete-pretty-hog.ngrok-free.app. Our new online platform is set to considerably ease access for researchers, particularly those from experimental backgrounds who may not have extensive computational support or specialized expertise, to our powerful m5C prediction tools. To use it, simply paste your RNA sequences (in FASTA format) into the input box on the web interface, as Figure 8 demonstrates.

This platform supports two flexible input methods: users can either directly upload RNA sequence files in FASTA format or choose to paste the sequence data directly into the provided text box, with an option to “Load Example Data” for familiarization with the required input format. Upon sequence submission, the server promptly executes the FusDRM-m5C prediction pipeline, presenting the results directly within the “Prediction Results” area. For improved readability and easier interpretation, predicted m5C sites are clearly color-coded according to their confidence levels: green for High, yellow for Medium, and red for Low. The platform also incorporates convenient search and item management functionalities. Additionally, users may opt to provide an email address to receive a copy of their prediction results.

## 4 Conclusion

This study introduces FusDRM-m5C, a novel deep learning framework for the accurate prediction of m5C sites from RNA sequences. A core contribution of the work is its multi-branch architecture that effectively integrates three distinct feature types: one-hot encoding, Z-curve-based geometrical features, and secondary structure. By utilizing Dilated Convolutional Neural Networks and a Multi-Head Self-Attention mechanism within each branch, the model captures multi-scale patterns and weighs context-dependent information from each feature type. These distinct high-level representations are then integrated via concatenation before being fed into fully connected layers, demonstrating outstanding performance and high robustness in both cross-validation and independent testing. Building on this, the application of STREME for cross-species motif analysis not only corroborated the model’s predictive capabilities but, more importantly, uncovered both conserved and species-specific motifs around m5C sites. These findings provide crucial biological insights into the model’s variable generalization performance across species and illuminate potential evolutionary divergences in m5C regulation.

Despite its excellent performance, several limitations of FusDRM-m5C should be acknowledged. First, the cross-species analysis highlighted that its generalization capability is influenced by species-specific features, indicating that patterns learned from one species may not be fully transferable to distantly related ones. Second, the model was trained and evaluated on balanced datasets. This approach was intentionally chosen to prevent the model from developing a bias towards the vastly more abundant negative class of non-m5C sites, thereby forcing it to learn the distinguishing features of true positive sites. However, this method does not reflect the naturally occurring distribution of m5C sites, which is highly imbalanced. Consequently, the model’s performance metrics, particularly precision, might be overestimated when applied to whole-transcriptome screening, where the false positive rate could be a more significant concern. Third, the model’s predictive power is constrained by the availability and reliability of training data, a limitation that directly impacts its practical utility across diverse research scenarios. While the model may perform robustly on data-rich model organisms, such as human and mouse, its prediction confidence is substantially diminished when applied to species that lack high-quality, large-scale datasets. Lastly, the model currently relies on features derived from the primary sequence and predicted secondary structure. This reliance on external tools for secondary structure prediction is an inherent vulnerability, as inaccuracies from these tools can propagate through the model and introduce errors. Beyond this, the model does not yet incorporate other potentially influential biological information, such as RNA tertiary structure or evolutionary conservation scores, which may further limit its predictive precision.

Future research should be directed at addressing these limitations. To enhance cross-species predictive capability, future work could explore advanced transfer learning strategies or explicitly integrate phylogenetic information into the model architecture. To better simulate real-world applications and address the data imbalance issue, models should be developed or evaluated on imbalanced datasets, potentially using techniques like focal loss or cost-sensitive learning. This would also involve assessing the current model on large-scale transcriptomic sequences to more accurately gauge its practical utility and false-positive rate. Additionally, to address potential errors introduced by external RNA structure prediction tools, future models could incorporate end-to-end architectures that learn structural representations directly from the sequence, thereby reducing dependency on pre-computed predictions. Another promising direction is to enrich the feature set by incorporating multi-omics data, such as RBP binding sites and gene expression profiles, could create more powerful prediction models. Finally, the species-specific motifs identified in this study warrant experimental validation to confirm their roles in m5C regulation and deepen the understanding of the biological significance of this modification. By systematically addressing these points, future iterations of the model are expected to achieve superior predictive accuracy and broader applicability in transcriptome-wide methylation studies.

## Data Availability

Publicly available datasets were analyzed in this study. The data and code for this study are available at: https://github.com/hhui0/FusDRM-m5C-code.

## References

[B1] Abbas Z. Rehman M. U. Tayara H. Lee S. W. Chong K. T. (2024). m5C-Seq: machine learning-enhanced profiling of RNA 5-methylcytosine modifications. Comput. Biol. Med. 182, 109087. 10.1016/j.compbiomed.2024.109087 39232403

[B2] Aslam I. Shah S. Jabeen S. M E. L. A A. A. L. Ul Haq N. (2023). A CNN based m5c RNA methylation predictor. Sci. Rep. 13 (1), 21885. 10.1038/s41598-023-48751-9 38081880 PMC10713599

[B3] Bai S. Kolter J. Z. Koltun V. (2018). An empirical evaluation of generic convolutional and recurrent networks for sequence modeling. arXiv Prepr. arXiv:1803.01271. 10.48550/arXiv.1803.01271

[B4] Bai L. Liu F. Wang Y. Su J. Liu L. (2025). MultiV_Nm: a prediction method for 2′-O-methylation sites based on multi-view features. Front. Genet. 16, 1608490–2025. 10.3389/fgene.2025.1608490 40496055 PMC12149135

[B5] Bailey T. L. (2021). STREME: accurate and versatile sequence motif discovery. Bioinformatics 37 (18), 2834–2840. 10.1093/bioinformatics/btab203 33760053 PMC8479671

[B6] Bailey T. L. Boden M. Buske F. A. Frith M. Grant C. E. Clementi L. (2009). MEME SUITE: tools for motif discovery and searching. Nucleic Acids Res. 37 (Web Server issue), W202–W208. 10.1093/nar/gkp335 19458158 PMC2703892

[B7] Bilal A. Alarfaj F. K. Khan R. A. Suleman M. T. Long H. (2025). m5c-iEnsem: 5-Methylcytosine sites identification through ensemble models. Bioinformatics 41 (1), btae722. 10.1093/bioinformatics/btae722 39657957 PMC11911556

[B8] Blanco S. Dietmann S. Flores J. V. Hussain S. Kutter C. Humphreys P. (2014). Aberrant methylation of tRNAs links cellular stress to neuro-developmental disorders. EMBO J. 33 (18), 2020–2039. 10.15252/embj.201489282 25063673 PMC4195770

[B9] Cappannini A. Ray A. Purta E. Mukherjee S. Boccaletto P. Moafinejad S. N. (2024). MODOMICS: a database of RNA modifications and related information. 2023 update. Nucleic Acids Res. 52 (D1), D239–D244. 10.1093/nar/gkad1083 38015436 PMC10767930

[B10] Chai D. Jia C. Zheng J. Zou Q. Li F. (2021). Staem5: a novel computational approachfor accurate prediction of m5C site. Mol. Ther. Nucleic Acids 26, 1027–1034. 10.1016/j.omtn.2021.10.012 34786208 PMC8571400

[B11] Chen X. Xiong Y. Liu Y. Chen Y. Bi S. Zhu X. (2020). m5CPred-SVM: a novel method for predicting m5C sites of RNA. BMC Bioinforma. 21 (1), 489. 10.1186/s12859-020-03828-4 33126851 PMC7602301

[B12] Dou L. Li X. Ding H. Xu L. Xiang H. (2020). Prediction of m5C modifications in RNA sequences by combining multiple sequence features. Mol. Ther. Nucleic Acids 21, 332–342. 10.1016/j.omtn.2020.06.004 32645685 PMC7340967

[B13] Edelheit S. Schwartz S. Mumbach M. R. Wurtzel O. Sorek R. (2013). Transcriptome-wide mapping of 5-methylcytidine RNA modifications in bacteria, archaea, and yeast reveals m5C within archaeal mRNAs. PLoS Genet. 9 (6), e1003602. 10.1371/journal.pgen.1003602 23825970 PMC3694839

[B14] Fang T. Zhang Z. Sun R. Zhu L. He J. Huang B. (2019). RNAm5CPred: prediction of RNA 5-Methylcytosine sites based on three different kinds of nucleotide composition. Mol. Ther. Nucleic Acids 18, 739–747. 10.1016/j.omtn.2019.10.008 31726390 PMC6859278

[B15] Feng P. Ding H. Chen W. Lin H. (2016). Identifying RNA 5-methylcytosine sites via pseudo nucleotide compositions. Mol. Biosyst. 12 (11), 3307–3311. 10.1039/c6mb00471g 27531244

[B16] Frommer M. McDonald L. E. Millar D. S. Collis C. M. Watt F. Grigg G. W. (1992). A genomic sequencing protocol that yields a positive display of 5-methylcytosine residues in individual DNA strands. Proc. Natl. Acad. Sci. U. S. A. 89 (5), 1827–1831. 10.1073/pnas.89.5.1827 1542678 PMC48546

[B17] Fu L. Niu B. Zhu Z. Wu S. Li W. (2012). CD-HIT: accelerated for clustering the next-generation sequencing data. Bioinformatics 28 (23), 3150–3152. 10.1093/bioinformatics/bts565 23060610 PMC3516142

[B18] Fu H. Ding Z. Wang W. (2025). Trans-m5C: a transformer-based model for predicting 5-methylcytosine (m5C) sites. Methods 234, 178–186. 10.1016/j.ymeth.2024.12.010 39742984

[B19] Gao F. Zhang C. T. (2004). Comparison of various algorithms for recognizing short coding sequences of human genes. Bioinformatics 20 (5), 673–681. 10.1093/bioinformatics/btg467 14764563

[B20] Gupta S. Stamatoyannopoulos J. A. Bailey T. L. Noble W. S. (2007). Quantifying similarity between motifs. Genome Biol. 8 (2), R24. 10.1186/gb-2007-8-2-r24 17324271 PMC1852410

[B21] Hasan M. M. Basith S. Khatun M. S. Lee G. Manavalan B. Kurata H. (2021). Meta-i6mA: an interspecies predictor for identifying DNA N6-methyladenine sites of plant genomes by exploiting informative features in an integrative machine-learning framework. Brief. Bioinform 22 (3), bbaa202. 10.1093/bib/bbaa202 32910169

[B22] Hasan M. M. Tsukiyama S. Cho J. Y. Kurata H. Alam M. A. Liu X. (2022). Deepm5C: a deep-learning-based hybrid framework for identifying human RNA N5-methylcytosine sites using a stacking strategy. Mol. Ther. 30 (8), 2856–2867. 10.1016/j.ymthe.2022.05.001 35526094 PMC9372321

[B23] He K. Zhang X. Ren S. Sun J. (2016). “Deep residual learning for image recognition,” in Proceedings of the IEEE conference on computer vision and pattern recognition, 770–778.

[B24] He Z. Xu J. Shi H. Wu S. (2022). m5CRegpred: epitranscriptome target prediction of 5-Methylcytosine (m5C) regulators based on sequencing features. Genes (Basel) 13 (4), 677. 10.3390/genes13040677 35456483 PMC9025882

[B25] Huang G. Liu Z. Van Der Maaten L. Weinberger K. Q. (2017). “Densely connected convolutional networks,” in Proceedings of the IEEE conference on computer vision and pattern recognition, 4700–4708.

[B26] Huang J. Wang X. Xia R. Yang D. Liu J. Lv Q. (2024). Domain-knowledge enabled ensemble learning of 5-formylcytosine (f5C) modification sites. Comput. Struct. Biotechnol. J. 23, 3175–3185. 10.1016/j.csbj.2024.08.004 39253057 PMC11381828

[B27] Hussain S. Sajini A. A. Blanco S. Dietmann S. Lombard P. Sugimoto Y. (2013). NSun2-mediated cytosine-5 methylation of vault noncoding RNA determines its processing into regulatory small RNAs. Cell Rep. 4 (2), 255–261. 10.1016/j.celrep.2013.06.029 23871666 PMC3730056

[B28] Jia J. Qin L. Lei R. (2023). im5C-DSCGA: a proposed hybrid framework based on improved DenseNet and attention mechanisms for identifying 5-methylcytosine sites in human RNA. Front. Biosci. Landmark Ed. 28 (12), 346. 10.31083/j.fbl2812346 38179749

[B29] Jiang D. Ao C. Li Y. Yu L. (2025). Feadm5C: enhancing prediction of RNA 5-Methylcytosine modification sites with physicochemical molecular graph features. Genomics 117 (3), 111037. 10.1016/j.ygeno.2025.111037 40127825

[B30] Kurata H. Harun-Or-Roshid M. Mehedi Hasan M. Tsukiyama S. Maeda K. Manavalan B. (2024). MLm5C: a high-precision human RNA 5-methylcytosine sites predictor based on a combination of hybrid machine learning models. Methods 227, 37–47. 10.1016/j.ymeth.2024.05.004 38729455

[B31] Lei R. Jia J. Qin L. (2025). im7G-DCT: a two-branch strategy model based on improved DenseNet and transformer for m7G site prediction. Comput. Biol. Chem. 118, 108473. 10.1016/j.compbiolchem.2025.108473 40245811

[B32] Li G. Yang Y. Li D. Su X. Zeng Z. Hu P. (2025). A bijective inference network for interpretable identification of RNA N6-methyladenosine modification sites. Pattern Recognit. 164, 111541. 10.1016/j.patcog.2025.111541

[B33] Linghui Q. Jie H. Guancheng W. Tao Y. Yanglin G. (2022). Promising novel biomarkers and candidate drugs or herbs in osteoarthritis: evidence from bioinformatics analysis of high‐throughput data. Curr. Bioinforma. 17 (5), 462–472. 10.2174/1574893617666220331090947

[B34] Liu Y. Shen Y. Wang H. Zhang Y. Zhu X. (2022). m5Cpred-XS: a new method for predicting RNA m5C sites based on XGBoost and SHAP. Front. Genet. 13, 853258. 10.3389/fgene.2022.853258 35432446 PMC9005994

[B35] Lorenz R. Bernhart S. H. Honer Zu Siederdissen C. Tafer H. Flamm C. Stadler P. F. (2011). ViennaRNA package 2.0. Algorithms Mol. Biol. 6, 26. 10.1186/1748-7188-6-26 22115189 PMC3319429

[B36] Lu R. Qiao J. Li K. Zhao Y. Jin J. Cui F. (2025). ERNIE-ac4C: a novel deep learning model for effectively predicting N4-acetylcytidine sites. J. Mol. Biol. 437 (6), 168978. 10.1016/j.jmb.2025.168978 39900287

[B37] Lv H. Zhang Z. M. Li S. H. Tan J. X. Chen W. Lin H. (2020). Evaluation of different computational methods on 5-methylcytosine sites identification. Brief. Bioinform 21 (3), 982–995. 10.1093/bib/bbz048 31157855

[B38] Ma J. Song B. Wei Z. Huang D. Zhang Y. Su J. (2022). m5C-Atlas: a comprehensive database for decoding and annotating the 5-methylcytosine (m5C) epitranscriptome. Nucleic Acids Res. 50 (D1), D196–D203. 10.1093/nar/gkab1075 34986603 PMC8728298

[B39] Malebary S. J. Alromema N. Suleman M. T. Saleem M. (2024). m5c-iDeep: 5-methylcytosine sites identification through deep learning. Methods 230, 80–90. 10.1016/j.ymeth.2024.07.008 39089345

[B40] Manavalan B. Hasan M. M. Basith S. Gosu V. Shin T. H. Lee G. (2020). Empirical comparison and analysis of web-based DNA N (4)-Methylcytosine site prediction tools. Mol. Ther. Nucleic Acids 22, 406–420. 10.1016/j.omtn.2020.09.010 33230445 PMC7533314

[B41] Masiello I. Biggiogera M. (2017). Ultrastructural localization of 5-methylcytosine on DNA and RNA. Cell Mol. Life Sci. 74 (16), 3057–3064. 10.1007/s00018-017-2521-1 28391361 PMC11107537

[B42] Qazi S. Shah D. Khan M. A. U. Ali S. Abrar M. Khan A. (2025). m5C‐TNKmer: identification of 5‐Methylated base cytosine of ribonucleic acid using supervised machine learning techniques. Eng. Rep. 7 (1), e13073. 10.1002/eng2.13073

[B43] Qiu W. R. Jiang S. Y. Xu Z. C. Xiao X. Chou K. C. (2017). iRNAm5C-PseDNC: identifying RNA 5-methylcytosine sites by incorporating physical-chemical properties into pseudo dinucleotide composition. Oncotarget 8 (25), 41178–41188. 10.18632/oncotarget.17104 28476023 PMC5522291

[B44] Roundtree I. A. Evans M. E. Pan T. He C. (2017). Dynamic RNA modifications in gene expression regulation. Cell 169 (7), 1187–1200. 10.1016/j.cell.2017.05.045 28622506 PMC5657247

[B45] Squires J. E. Patel H. R. Nousch M. Sibbritt T. Humphreys D. T. Parker B. J. (2012). Widespread occurrence of 5-methylcytosine in human coding and non-coding RNA. Nucleic Acids Res. 40 (11), 5023–5033. 10.1093/nar/gks144 22344696 PMC3367185

[B46] Tanaka E. Bailey T. Grant C. E. Noble W. S. Keich U. (2011). Improved similarity scores for comparing motifs. Bioinformatics 27 (12), 1603–1609. 10.1093/bioinformatics/btr257 21543443 PMC3106196

[B47] Tang Y. Chen K. Song B. Ma J. Wu X. Xu Q. (2021). m6A-Atlas: a comprehensive knowledgebase for unraveling the N6-methyladenosine (m6A) epitranscriptome. Nucleic Acids Res. 49 (D1), D134–D143. 10.1093/nar/gkaa692 32821938 PMC7779050

[B48] Vaswani A. Shazeer N. Parmar N. Uszkoreit J. Jones L. Gomez A. N. (2017). Attention is all you need. Adv. Neural Inf. Process. Syst. 30. 10.48550/ARXIV.1706.03762

[B49] Xu H. Hu R. Jia P. Zhao Z. (2020). 6mA-Finder: a novel online tool for predicting DNA N6-methyladenine sites in genomes. Bioinformatics 36 (10), 3257–3259. 10.1093/bioinformatics/btaa113 32091591 PMC7214014

[B50] Yang X. Yang Y. Sun B. F. Chen Y. S. Xu J. W. Lai W. Y. (2017). 5-methylcytosine promotes mRNA export - NSUN2 as the methyltransferase and ALYREF as an m(5)C reader. Cell Res. 27 (5), 606–625. 10.1038/cr.2017.55 28418038 PMC5594206

[B51] Yu F. Koltun V. (2015). Multi-scale context aggregation by dilated convolutions. arXiv Prepr. arXiv:1511.07122. 10.48550/arXiv.1511.07122

[B52] Zaccara S. Ries R. J. Jaffrey S. R. (2023). Publisher correction: reading, writing and erasing mRNA methylation. Nat. Rev. Mol. Cell Biol. 24 (10), 770. 10.1038/s41580-023-00654-3 37604996

[B53] Zhang R. Zhang C. T. (1994). Z curves, an intutive tool for visualizing and analyzing the DNA sequences. J. Biomol. Struct. Dyn. 11 (4), 767–782. 10.1080/07391102.1994.10508031 8204213

[B54] Zhou Y. Wu J. Yao S. Xu Y. Zhao W. Tong Y. (2023). DeepCIP: a multimodal deep learning method for the prediction of internal ribosome entry sites of circRNAs. Comput. Biol. Med. 164, 107288. 10.1016/j.compbiomed.2023.107288 37542919

